# Cutaneous Metastasis from Colorectal Cancer: Making Light on an Unusual and Misdiagnosed Event

**DOI:** 10.3390/life11090954

**Published:** 2021-09-11

**Authors:** Paola Parente, Davide Ciardiello, Luca Reggiani Bonetti, Vincenzo Famiglietti, Gerardo Cazzato, Stefania Caramaschi, Vito Attino, Diego Urbano, Giuseppe Di Maggio, Giuseppe Ingravallo

**Affiliations:** 1 Pathology Unit, Fondazione IRCCS Ospedale Casa Sollievo della Sofferenza, 71013 San Giovanni Rotondo, Italy; v.attino@operapadrepio.it (V.A.); urbediego@libero.it (D.U.); giuseppedimaggio12@yahoo.it (G.D.M.); 2 Oncologia Medica, Dipartimento di Medicina di Precisione, Università degli Studi della Campania “Luigi Vanvitelli”, 80138 Napoli, Italy; davideciardiello@yahoo.it (D.C.); vincenzo.famiglietti@yahoo.it (V.F.); 3 Unità di Oncologia, Fondazione IRCCS Ospedale Casa Sollievo della Sofferenza, 71013 San Giovanni Rotondo, Italy; 4 Department of Medical and Surgical Sciences for Children and Adults, University of Modena and Reggio Emilia-AOU Policlinico of Modena, 41124 Modena, Italy; luca.reggianibonetti@unimore.it (L.R.B.); stefania.caramaschi@unimore.it (S.C.); 5 Pathology Unit, Department of Organ Transplantation and Emergency (DETO), University Hospital of Bari, 70124 Bari, Italy; gerycazzato@hotmail.it (G.C.); giuseppe.ingravallo@uniba.it (G.I.)

**Keywords:** adenocarcinoma, colorectal cancer, cutaneous metastasis, intestinal cancer, skin tumors, visceral metastases

## Abstract

Cutaneous metastasis from solid tumors is a rare event and usually represents a late occurrence in the natural history of an advanced visceral malignancy. Rarely, cutaneous metastasis has been described in colorectal cancer patients. The most frequent cutaneous site of colorectal metastasis is the surgical scar in the abdomen following the removal of the primary malignancy, followed by the extremities, perineum, head, neck, and penis. Metastases to the thigh and back of the trunk are anecdotical. Dermatological diagnosis of cutaneous metastasis can be quite complex, especially in unusual sites, such as in the facial skin or thorax and in cases of single cutaneous lesions since metastasis from colorectal cancer is not usually the first clinical hypothesis, leading to misdiagnosis. To date, due to the rarity of cutaneous metastasis from colorectal cancer, little evidence, most of which is based on case reports and very small case series, is currently available. Therefore, a better understanding of the clinic-pathological characteristics of this unusual metastatic site represents an unmet clinical need. We present a large series of 29 cutaneous metastases from colorectal cancer with particular concerns regarding anatomic localization and the time of onset with respect to primitive colorectal cancer and visceral metastases.

## 1. Introduction

Cutaneous metastasis from solid tumors is rare and occurs in 0.7% to 5% of all cancers [1]. Cutaneous metastasis generally represents a late event of an advanced visceral malignancy and occurs with greater frequency in melanoma and lung carcinoma, followed by kidney and ovary cancer [1,2]. Cutaneous metastasis has rarely been described in hepato-biliary malignancies [3]. 

Colorectal cancer (CRC) typically metastasizes to the lymph nodes, lung, liver, and peritoneum, and the development of skin metastasis from CRC is uncommon (2.3% to 6%) [2]. The most frequent cutaneous site of CRC metastasis is the surgical scar in the abdomen resulting from the removal of the primary malignancy, followed by the extremities, perineum, head, neck, and penis. An atypical localization is the umbilical region also knows as ‘Sister Mary Joseph nodule’ [4]. Metastases to the thigh and back of the trunk are anecdotical [4,5]. 

The literature reports two distinct clinical patterns of skin metastases from CRC: the first showing multiple visceral and cutaneous metastases at the time of presentation and the second developing cutaneous metastases during the follow-up, after the resection of the primary tumor [6]. 

Several clinical presentations have been described in CRC cutaneous metastasis, the most frequent being ulcers, papules, nodules, plaques, and rapidly growing painless dermal or subcutaneous nodules with intact overlying epidermis or mimic inflammatory dermatosis [5]. 

The diagnosis of cutaneous metastasis can be quite complex, especially in unusual sites, such as in the facial skin or thorax, and in cases of single cutaneous lesions, since metastasis from CRC usually is not the first clinical hypothesis, mostly due to the absence of adequate clinical context reporting CRC notice.

To date, due to the rarity of cutaneous metastasis from CRC, little evidence, most of which is represented by case reports and very small case series, is currently available. Therefore, a better understanding of the clinic-pathological characteristics of this unusual metastatic site represents an unmet clinical need. We have retrospectively analyzed a large series of 29 cutaneous metastases from CRC and their epidemiological and clinical features with particular concerns regarding anatomic localization and the time of onset with respect to the primitive CRC and visceral metastases. 

## 2. Materials and Methods

We retrospectively collected 29 cases of cutaneous metastasis from CRC from the electronic pathology archives at the Unit of Pathology, Fondazione IRCCS Ospedale Casa Sollievo della Sofferenza, San Giovanni Rotondo, Italy, at the Unit of Pathology, University of Bari, Italy, and at the Unit of Pathology, Modena, Italy, between 2004 and 2020. 

All information regarding human tissue was managed using anonymous numerical codes, and all samples were handled in compliance with the Helsinki Declaration (https://www.wma.net/what-we-do/medical-ethics/declaration-of-helsinki/ (accessed on 10 September 2021)).

### Clinic-Pathologic Evaluation

The following data were collected: demographic data (age at diagnosis of skin metastasis, gender), anatomic localization, number (single/multiple) and gross findings (size and morphologic features) of the lesions, clinical hypothesis of the entity (primitive skin tumour, dermatosis, metastasis), time of onset with respect to CRC diagnosis (synchronous if ≤12 months and metachronous if >12 months from CRC diagnosis, respectively), time of onset with respect to visceral metastases (synchronous if ≤12 months and metachronous if >12 months from visceral metastases diagnosis and ‘previous’ if skin metastases arose before visceral metastases), histotype according to World Health Organization (WHO) Classification of Digestive System Tumours, 5th edition, 2019 [7], and tumor staging based on surgery according to the Union for International Cancer Control/American Joint Committee on Cancer (UICC/AJCC) 8th edition [8] for primitive CRC (Table 1).

The morphology of the collected tumor samples was revised, and new sections were microtome cut and stained with Haematoxylin and Eosin (H&E) when necessary, initially by the submitting center and subsequently by the central collection center (Fondazione IRCCS Ospedale Casa Sollievo della Sofferenza, San Giovanni Rotondo). The following histological parameters of cutaneous metastases were collected: (i) localization of the metastasis (dermal, ipodermal, dermo-ipodermal), (ii)differentiation grading (based on percentage of glandular component according to World Health Organization (WHO) Classification of Digestive System Tumours, 5th edition, 2019 [7]), and (iii) additional morphological findings (squamous component; mucinous component; signet ring component) (Table 2). 

## 3. Results

### 3.1. Clinical Findings

**Figure 1:**
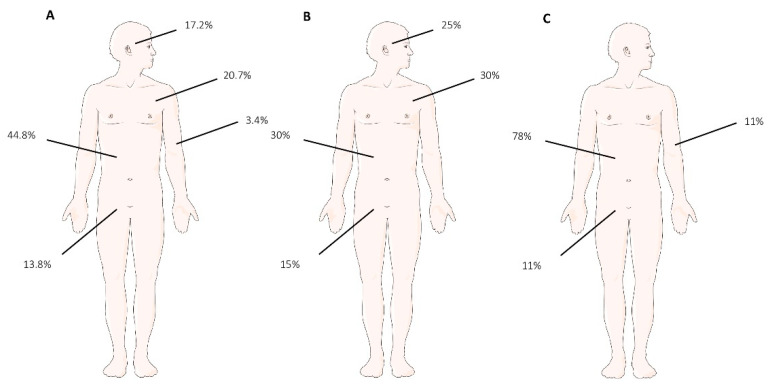
(**A**) Localization and percentage of cutaneous metastasis: abdomen 13/29 (44.8%); thorax 6/29 (20.7%); facial skin 5/29 (17.2%); pelvis 4/29 (13.8%); arm 1/29 (3.4%). (**B**) Localization and percentage of single lesions: abdomen 6/20 (30%); thorax 6/20 (30%); facial skin 5/20 (25%); pelvis 3/20 (15%). (**C**) Localization and percentage of multiple lesions: abdomen 7/9 (30%), pelvis 1/29 (11%), arm 1/9 (11%).

Of the 29 patients, the median age was 72 years of age (range 41–89); most of the patients were male (20 males, 9 females). Out of the reported cases, 20 out of 29 cases presented as a single skin lesion (70%), 6 of which occurring in the abdomen (6/20; 30%), 6 occurring in the thoracic skin (6/20; 30%), 3 occurring in the perineum (3/20; 15%), and 5 occurring in the facial skin (5/20; 25%). A total of 9 out of 29 (30%) patients presented multiple skin lesions, 7 of which occurred in the abdomen (7/9; 78%), 1 of which occurred in the perineum (1/9; 11%), and 1 of which occurred in the arm (1/9; 11%). A total of 13 out of 29 cases (45%) were localized in the abdominal skin; 6/29 cases (20%) were localized in the thoracic skin; 5/29 cases (17%) on facial skin; 4/29 cases (13%) were localized in the perineum; and 1/29 case (5%) was localized on the arm (Figure 1). 

The median size of the lesions was 2.3 cm (range 0.4–8) (Table 2). A total of 15 cases (15/29, 52%) were synchronous, and 14/29 cases (48%) were metachronous to CRC. In the synchronous metastases, 8/15 were located in the abdomen (53%), 4/15 (26%) were located in the thoracic skin, 2/15 (13%) were located in the facial skin, and 1/15 (8%) were located on the arm, respectively. In the metachronous metastases, 5/14 (36%) were located in the abdomen, 2/14 (14%) were located in the thoracic skin, 4/14 (28%) were located in the perineum, and 3/14 (22%) were located in the facial skin (Table 3).

A total of 5/29 cases (17%) were metachronous to visceral metastases from CRC; 3/29 cases (10%) presented before, and 7/29 cases (24%) were synchronous to visceral metastases, respectively. In those that were synchronous with respect to visceral metastases, 2/7 cases (28.5%) were located in the abdomen, 2/7 (28.5%) cases were located in the thoracic skin, 2/7 (28.5%) cases were located in the perineum, and 1/7 case (15%) was located in the arm. In the metachronous lesions, 2/5 cases (40%) were located in the abdomen, 2/5 cases (40%) were located in the thorax, and 1/5 (20%) was located in the facial skin. Interestingly, of the three cases arising before visceral metastases, one case was in the abdomen, one case was in the thoracic skin, and one case was in the facial skin. In 14/29 cases (49%), no data regarding visceral metastases were available (Table 4).

Based on presentation and according to the medical history, twenty cases (20/29, 70%) were clinically referred as suspected of CRC metastases; instead, 4/29 cases (13%) were submitted as primitive cutaneous tumors. In 5/29 cases (17%), no clinical hypothesis was referred (Table 2). Regarding the clinical presentation, twenty-four cases (83%) presented macroscopically as ‘nodular lesions’; 4/29 (14%) presented as ‘vegetant’ lesions, and 1/29 case (3%) appeared as an ipodermal cystic lesion. A total of 6/29 lesions (20%) were ulcerated. In 21/29 cases (72%), we were able to acquire the pathological stage of CRC, whereas in 8/29 cases (28%), we were not able to determine the pathological stage of CRC, as the laboratory services did not inform us. In 13/29 patients (45%), primary CRC was located in right colon; in 9/29 cases (31%), it was located in the left colon, in 5/29 (17%), it was located in the rectum, and in 1/29 cases, (3.5%) it was located in the transverse colon, and in 1/29 cases (3.5%), it was located in the anus (Table 1).

### 3.2. Histopathological Findings

**Figure 2:**
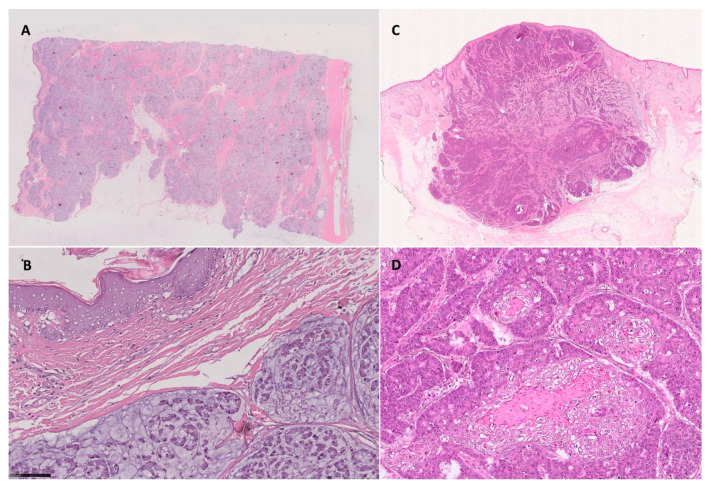
(**A**,**B**) Mucinous component in cutaneous metastasis from CRC ((**A**) H&E 0.5X; (**B**) H&E 25X). (**C**,**D**) Squamous component in facial skin metastasis from adenosquamous CRC ((**C**) H&E 0.5X; (**D**) H&E 20X).

Morphologic findings of the cutaneous metastases are described in Table 2. Twenty-seven cases (93%) presented as dermal/ipodermal lesions, and two cases (7%) were dermal-confined lesions. For 22 patients (76%), we were able to obtain a histological surgical sample of primitive CRC. Of them, 9/22 cases (40%) were low-grade adenocarcinoma (i.e., G1-G2 sec WHO 2019), and 13/22 tumors (60%) were high grade adenocarcinoma (i.e., G3 sec WHO 2019). Moreover, for 28 tumor samples, we could assign grading to the cutaneous metastases. Of them, 14/28 (50%) were low-grade adenocarcinoma (i.e., G1-G2 sec WHO 2019), whereas 14/28 cases (50%) were high-grade adenocarcinoma (i.e., G3 sec WHO 2019). In one case, we could not evaluate a glandular component (i.e., grading) due to the small size of the biopsy. In 6/29 cases of the cutaneous samples, (20%), we described a mucinous and/or signet ring neoplastic component, and in 2/29 (6%), we were able to describe a squamous neoplastic component (Figure 2).

The histotype comparison between primitive CRC and the skin metastases is reported in Table 5, based on WHO classification [7]. Twenty-two cases of the surgical resections were classified as NOS-CRC; in three cases, we observed a mucinous-signet ring histotype; in two cases, we described an adenosquamous (ASC) histotype; in one case, a mucinous histotype was described, and in one case, a mucinous component was described (i.e., mucinous component less than 30%), respectively. All cases of skin metastases were classified as NOS-ADK; in five cases, we observed an additional mucinous component; in two cases, we observed a squamous component, and in the last one, a signet-ring-mucinous component was observed. In six out of eight skin metastases (75%), we observed the same histotype in both the primitive and cutaneous metastases; in detail, we observed two cases with the ASC histotype and four cases with mucinous component. In one case, the mucinous component observed in the primitive CRC was not evaluable in the skin metastases; in one case, we observed a mucinous component in a metastasis not described in primitive CRC.

## 4. Discussion

Cutaneous cancer metastases are a rare event, occurring in about 1.3% of cases at the time of presentation of the primary tumor [4]. The insurgence of cutaneous metastasis is infrequently described as the first sign of malignancy in CRC and occurs with greater frequency in breast and lung carcinoma, followed by kidney and ovarian cancer [1,5,9,10]. Moreover, cutaneous metastasis arising before visceral metastasis is an extremely unusual occurrence in CRC [4]. Therefore, due to the uncommon nature of this entity, the correct identification of cutaneous metastasis with dermoscopy is very hard, leading to misdiagnosis and underreporting of cutaneous metastasis clinical suspicion [11].

Very little is known about timing correlation between cutaneous metastases and CRC onset and visceral metastases from CRC onset, due to only case report about number and anatomical localization of skin metastases from CRC reported in Literature.

In current scenario, we described a series of 29 cases of cutaneous metastases from CRC with special concerns regarding the number, anatomical localization, and time of onset with respect to CRC diagnosis and visceral metastases. Moreover, some considerations regarding the histological features were discussed. 

In line with previous findings, our results confirm that the most frequent anatomic localizations of cutaneous metastasis resulting from CRC is abdomen followed by thoracic, facial skin, and perineum localization [2,4]. Interestingly, we observed that almost half of the cases are synchronous respect to CRC onset and that almost half are synchronous to visceral metastases. Furthermore, the most frequent presentation pattern of cutaneous metastases that was observed is represented by a single lesion (70%) compared to multiple lesions (30%). Remarkably, all facial skin and thoracic metastases arose as single lesions, and this finding might mislead the clinical hypothesis. In fact, clinical suspicion of metastasis resulting from CRC was only raised in two out of five cases of lesions with facial skin localization. In the other three cases, primitive cutaneous tumors (epithelioma and adnexal tumor) and no clinical suspicions were reported. In four out of six lesions with thoracic localization, clinical suspicion of metastases was reported; in the other two cases, no clinical hypothesis was advanced. Moreover, two cases of facial skin lesions were synchronous to CRC, with clinical presentation as epithelioma in one case and without clinical suspicion in the second case. Four cases of thoracic localization were synchronous to CRC, only two of which being hypothesized as metastasis from CRC, whereas in the remaining two cases, no clinical information was available. Interestingly, of the three facial skin metastases from CRC described in the literature [6,12,13], only one was synchronous to CRC diagnosis [12]. Of note, no study concerning thoracic cutaneous localization from CRC has been reported. Thus, these findings underline the importance of ensuring that an in-depth clinical information is requestion when submitting cutaneous lesions for histological evaluation. In fact, a single lesion is not usually clinically suspected of metastasis, and primitivity is suspected close to the anatomic localization of cutaneous lesions, such as lung cancer and breast lesions in case of thoracic localization.

Localization and the time of onset of cutaneous metastasis from CRC with respect to visceral metastases have been never described in detail. Skin metastases have only been described in a synchronous or metachronous timeframe with respect to visceral metastases, and in patients with no visceral metastases, skin lesions were found exclusively on the abdominal wall or perineum [6,12]. Intriguingly, of our 15 patients with visceral metastases notice, 3 cases of cutaneous metastases arose before visceral metastases, 7 were synchronous, and 5 were metachronous to visceral metastases. One of three cases arising before visceral metastasis was located in facial skin, and one in the thoracic skin, respectively. 

Finally, from a histological point of view, peculiar aspects of the cutaneous metastases described in this study should alert pathologists. In fact, we observed a squamous cheratinizant malignant component in a single lesion in facial skin in a patient without oncological anamnesis. The squamous component can be observed in primitive skin tumors, either epithelial and/or adnexal, confounding diagnosis. In our series, all cases with a mucinous and/or signet ring component were located in abdominal skin. Being that single cutaneous metastases often favor the closest primitivity [4], in a single thoracic lesion with a mucinous component at histological examination, a pulmonary primitivity can be more reasonably suspected with respect to CRC. In these cases, immunohistochemistry is useless because of the same immunophenotype documented in both pulmonary and CRC primitivity (i.e., TTF1 negativity, CK7 negativity, CK20 positivity, CDX2 positivity). Finally, half of the skin lesions were reported as undifferentiated, suggesting the aggressiveness of these tumors.

These results underline the need of an accurate compilation of histological requests and for a deep dermatological evaluation of patients who are undergoing screening or who have already been diagnosed with cancer in order to provide a high level of suspicion regarding the onset of cutaneous lesions, even if they are clinically compatible with benign illnesses. This evaluation also helps to determine appropriate therapy, because cutaneous metastases indicate an advanced and aggressive disease.

Our study has several limitations. Unfortunately, we were not able to study biological profiles of all of the primitive and metastatic lesions to suggest hypothesis regarding the different metastatic potential in different CRC histotypes and time of metastases onset. Further studies are needed to investigate the molecular landscape of CRC-related cutaneous metastasis. Additionally, due to the reduced number of patients and due to the retrospective nature of the study, it is very difficult to find any statistical associations between the histological features of primitive CRC (such as perineural invasion, tumor budding, pattern of invasion, macroscopic appearance) and the correlation with other risk factor (e.g., exposure to sun, smoke, and alcohol consumption).

## 5. Conclusions

In conclusion, cutaneous metastasis from CRC is a rare but underreported phenomenon, which should not be ignored, as it indicates an advanced and aggressive disease. Cutaneous metastases from CRC could be the first sign of tumor metastases arising before visceral localization and frequently present as ‘single’ lesions and at an unusual site. Any change in the skin should raise the suspicion of metastases in the correct clinical context, and biopsy is mandatory in patients with a history of cancer. Early diagnosis will be the key element for adequate management, which requires careful physical examination and a carefully filled format for histological examination. Larger prospective studies are required to better understand the biology of cutaneous metastasis.

## Figures and Tables

**Table 1:** Clinic-pathological characteristics of primitive CRC. NOS: not otherwise specified (according to World Health Organization (WHO) Classification of Digestive System Tumours, 5th edition, 2019 [7]); NE: not evaluable.

Case	Age (Years)	Gender	Tumor Location	Histotype	Grading	Pathological Stage UICC 2017
1	70	Male	Left colon	Adenosquamous	G3	pT4a pN2a
2	41	Male	Right colon	Signet ring; mucinous	G3	NE
3	60	Male	Left colon	NOS	G3	pT4a pN2b
4	70	Female	Right colon	NOS	G2	pT3 pN0
5	85	Male	Left colon	NOS	G3	pT4a pN1b
6	48	Male	Right colon	NOS	G2	pT4a pN0
7	76	Female	Right colon	NOS	G2	pT4a pN1
8	74	Male	Left colon	NOS with mucinous component	NE	pT3 pN0
9	70	Male	Right colon	NOS	NE	NE
10	74	Male	Right colon	NOS	NE	NE
11	84	Female	Left colon	NOS	G2	pT3 pN0
12	63	Male	Left colon	NOS	NE	NE
13	62	Male	Rectum	NOS	NE	NE
14	63	Male	Rectum	NOS	NE	pT3 pN2
15	69	Female	Anus	NOS	NE	NE
16	74	Male	Right colon	Adenosquamous	G3	pT4a pN2b
17	76	Male	Rectum	NOS	G2	pT3 pN0
18	89	Female	Right colon	NOS	G3	pT3 pN1p M1
19	79	Male	Right colon	NOS	G3	pT4 pN1b M1
20	73	Male	Sigma-rectum	NOS	G2	pT3 N1b
21	69	Female	Transverse colon	NOS	G2	pT3 N1b M1
22	77	Male	Right colon	NOS	G2	pT4a N2
23	80	Male	Rectum	NOS	G3	pT4 N0
24	79	Female	Right colon	NOS	G3	NE
25	75	Male	Right colon	Signet ring; mucinous	G3	pT4 N2 M1
26	66	Female	Right colon	Signet ring; mucinous	G3	pT4 N2
27	83	Male	Left colon	NOS	G3	pT3 N1
28	85	Male	Left colon	NOS	G3	pT1 N0
29	83	Female	Left colon	NOS	G2	NE

**Table 2:** Clinic-pathological characteristics of the skin CRC metastases. CM: centimeters; NE: not evaluable; ADK NOS: adenocarcinoma not otherwise specified [7].

Case	Macroscopy	Size (CM)	Clinical Hypothesis	Localization	Grading of Skin Metastasis (Glandular Component)	Additional Morphological Findings of Skin Metastasis [7]
1	Nodular lesion	2	Epithelioma	Dermo-ipodermal	G3	Keratinizant-squamous component
2	Nodular lesion	1.3	Metastasis	Dermo-ipodermal	G3	ADK; NOS
3	Nodular lesion	3	Metastasis	Dermo-ipodermal	G3	Mucinous; signet ring
4	Vegetant ulcerating lesion	8	Metastasis	Dermo-ipodermal	G1	ADK; NOS
5	Ipodermal cystic lesion	2.5	NE	Dermo-ipodermal	G3	ADK; NOS
6	Vegetant ulcerating lesion	1	NE	Dermo-ipodermal	G2	ADK; NOS
7	Nodular lesion	2.5	NE	Dermo-ipodermal	G2	ADK; NOS
8	Nodular lesion	1.3	NE	Dermo-ipodermal	G1	Mucinous component
9	Nodular lesion	0.4	NE	Dermo-ipodermal	G3	ADK; NOS
10	Nodular lesion	1	Metastasis	Dermo-ipodermal	G1	Mucinous component
11	Nodular lesion	0.7	Metastasis	Dermal	G2	ADK; NOS
12	Nodular lesion	0.6	Metastasis	Dermo-ipodermal	G2	ADK; NOS
13	Nodular lesion	1.5	Metastasis	Dermo-ipodermal	G1	ADK; NOS
14	Nodular ulcerating lesion	2.5	Metastasis	Dermo-ipodermal	G1	ADK; NOS
15	Nodular lesion	8	Metastasis	Dermo-ipodermal	G3	Mucinous component
16	Nodular lesion	5	Metastasis	Dermo-ipodermal	G3	ADK; NOS
17	Nodular lesion	2.7	Metastasis	Dermo-ipodermal	G2	Nonkeratinizant squamous component
18	Nodular lesion	0.8	Adnexal tumor	Dermo-ipodermal	G3	ADK; NOS
19	Nodular lesion	1.5	Sebaceous cist	Dermal	G3	ADK; NOS
20	Nodular lesion	1	Metastasis	Dermo-ipodermal	G2	ADK; NOS
21	Nodular lesion	2	Metastasis	Dermo-ipodermal	G1	ADK; NOS
22	Nodular lesion	5	Metastasis	Dermo-ipodermal	G2	ADK; NOS
23	Nodular lesion	2.2	Metastasis	Dermo-ipodermal	G3	ADK; NOS
24	Nodular ulcerating lesion	1	Epithelioma	Dermo-ipodermal	G3	ADK; NOS
25	Nodular lesion	1.5	Metastasis	Dermo-ipodermal	NV	Mucinous
26	Nodular lesion	1	Metastasis	Dermo-ipodermal	G3	Mucinous
27	Nodular lesion	2.5	Metastasis	Dermo-ipodermal	G3	ADK; NOS
28	Vegetant ulcerating lesion	2	Metastasis	Dermo-ipodermal	G3	ADK; NOS
29	Nodular lesion	3	Metastasis	Dermo-ipodermal	G2	ADK; NOS

**Table 3:** Temporal correlation of cutaneous metastases with respect to CRC.

Localization	Synchronous to CRC	Metachronous to CRC	Total
Abdomen	8	5	13
Thorax	4	2	6
Facial Skin	2	3	5
Pelvis	0	4	4
Arm	1	0	1
	15	14	29

**Table 4:** Temporal correlation of cutaneous metastases with respect to visceral metastasis from CRC. VM: visceral metastasis.

Localization	Synchronous to VM	Onset before to VM	Metachronous to VM	Total
Abdomen	2	1	2	5
Thorax	2	1	2	5
Facial Skin	0	1	1	2
Pelvis	2	0	0	2
Arm	1	0	0	1
	7	3	5	15

**Table 5:** Comparison between the histology of the primary tumor and skin metastases. ADS: adenosquamous; NOS: not otherwise specified; ADK: adenocarcinoma.

Case	Primary Tumor Hystotype According to WHO 2019	Additional Morphological Findings of Skin Metastasis
1	ADS	Keratinizant squamous component
2	Signet ring; mucinous	ADK, NOS
3	Mucinous	Mucinous-signet ring
4	NOS	ADK, NOS
5	NOS	ADK, NOS
6	NOS	ADK, NOS
7	NOS with mucinous component	Mucinous component
8	NOS	ADK, NOS
9	NOS	ADK, NOS
10	NOS	Mucinous component
11	NOS	ADK, NOS
12	NOS	ADK, NOS
13	NOS	ADK, NOS
14	NOS	Mucinous component
15	NOS	ADK, NOS
16	ADS	Nonkeratinizant squamous component
17	NOS	ADK, NOS
18	NOS	ADK, NOS
19	NOS	ADK, NOS
20	NOS	ADK, NOS
21	NOS	ADK, NOS
22	NOS	ADK, NOS
23	NOS	ADK, NOS
24	NOS	ADK, NOS
25	Signet ring; mucinous	Mucinous component
26	Signet ring; mucinous	Mucinous component
27	NOS	ADK, NOS
28	NOS	ADK, NOS
29	NOS	ADK, NOS

## Data Availability

Not applicable.

## References

[1] Faenza M., Del Torto G., Di Costanzo P., Pieretti G., Lamberti R., Franco R., Ferraro G.A., Nicoletti G.F. (2019). Large single cutaneous metastasis of colon adenocarcinoma mimicking a squamous cell carcinoma of the skin: A case report. Int. J. Surg. Case Rep..

[2] Wang D.Y., Ye F., Lin J.J., Xu X. (2017). Cutaneous metastasis: A rare phenomenon of colorectal cancer. Ann. Surg. Treat. Res..

[3] Cazzato G., Colagrande A., Cimmino A., De Marco A., Romita P., Foti C., Resta L., Ingravallo G. (2021). Cutaneous Metastases from Primary Liver Cancers: The Need for Knowledge and Differential Diagnosis. Life.

[4] Nambiar S., Karippot A. (2018). Multiple Cutaneous Metastases as Initial Presentation in Advanced Colon Cancer. Case Rep. Gastrointest. Med..

[5] Bin Wong C.Y., Kalb R.E., Zeitouni N.C., Helm M.A., Helm T.N. (2013). The presentation, pathology, and current management strategies of cutaneous metastasis. N. Am. J. Med. Sci..

[6] Aravind B., Kumar R., Basnyat P. (2013). Cutaneous metastases secondary to colorectal carcinoma may not be as ominous as previously thought: A case report and review of the literature. BMJ Case Rep..

[7] World Health Organization (WHO) (2019). Classification of Digestive Tumours.

[8] Amin M.B. (2017). AJCC Cancer Staging Manual.

[9] Seyfried T.N., Huysentruyt L.C. (2013). On the Origin of Cancer Metastasis. Crit. Rev. Oncog..

[10] Krathen R.A., Orengo I.F., Rosen T. (2003). Cutaneous Metastasis: A Meta-Analysis of Data. South. Med. J..

[11] Chernoff K.A., Marghoob A.A., Lacouture M.E., Deng L., Busam K.J., Myskowski P.L. (2014). Dermoscopic Findings in Cutaneous Metastases. JAMA Dermatol..

[12] Parente P., Covelli C., Parrella P., Latiano T.P., Fiordelisi F., Pellico M.T., Maiello E., Graziano P. (2020). Intestinal adenosquamous carcinoma with a synchronous skin metastasis: A immunohistochemical and molecular analysis. Int. J. Color. Dis..

[13] Hashimi Y., Dholakia S. (2013). Facial cutaneous metastasis of colorectal adenocarcinoma. BMJ Case Rep..

